# An Overview of Parkinson's Disease: Curcumin as a Possible Alternative Treatment

**DOI:** 10.7759/cureus.25032

**Published:** 2022-05-15

**Authors:** Arjun Patel, Catherine A Olang, Gregory Lewis, Kesava Mandalaneni, Nikhilesh Anand, Vasavi Rakesh Gorantla

**Affiliations:** 1 Anatomical Sciences, St. George's University School of Medicine, St. George's, GRD; 2 Physiology, St. George's University School of Medicine, St. George's, GRD; 3 Pharmacology, American University of Antigua, St. John's, ATG; 4 Anatomical Sciences, St. George's University, St. George's, GRD

**Keywords:** neurodegenerative diseases., dopamine precursors, neuroprotective effects, neuroplasticity, curcuma longa, parkinson's disease

## Abstract

Parkinson's disease (PD) is a neurodegenerative disorder characterized by the loss of dopaminergic neurons in the substantia nigra of the midbrain and basal ganglia, followed by dopamine deficiency in the brain. Dopamine plays a crucial role in motor coordination, memory, and cognition; its decrease in PD leads to dyskinesia, cognitive deficits, and depression. In addition, the formation of alpha-synuclein protein aggregates (Lewy bodies) causes further damage to the CNS. Current treatment options include dopamine precursors, inhibitors of dopamine metabolism, upregulation of autophagy, adenosine A2A antagonists, and surgical intervention as a last resort. A challenge arises from a progressive decrease in treatment efficacy as the disease progresses and this necessitates exploration of adjunctive treatments. Epidemiological studies suggest that the prevalence of PD varies between ethnic groups of Caucasians, Asians, and African Americans. Notably, the prevalence of PD is lower in countries of Southeastern Asia including India. The differences in the diet of various ethnic groups may suggest an origin for this difference in the prevalence of PD. One staple ingredient in traditional Asian cuisine is turmeric. *Curcuma longa*, popularly known as turmeric, is an orange tuberous rhizome that has been used for centuries in traditional Indian cuisine and traditional medicine. Turmeric contains curcumin, a potent antioxidant that scavenges reactive oxygen species and chelates toxic metals. Curcumin has been proposed to be a neuroprotective agent due to its potent antioxidative properties. Though preliminary studies in animal model systems have suggested a protective effect of curcumin on dopaminergic neurons, the direct benefits of curcumin on the progress of PD remains poorly understood. In this review, we explore the promising use of curcumin as an adjunct to conventional PD treatments in order to enhance treatment and improve outcomes.

## Introduction and background

After Alzheimer's disease, Parkinson's disease (PD) is the second most common age-related neurodegenerative disease [1]. A PD diagnosis can be devastating for the person who has it and the family, who often would also be the caregivers. Moreover, despite the surgical and pharmacological interventions, the patient's physical and mental health declines from a certain period after the onset of the disease.

PD is characterized by loss of dopamine due to dysfunctional dopaminergic neurons and can be classified as a hypokinetic disorder. Dopamine is not only directly involved in movement and cognition but also plays a broad role in many other nervous system processes. Therefore, the loss of dopamine can lead to a broad range of sometimes severe neuropsychological symptoms, including motor defects, cognitive impairment, and depression. The PD progression is divided into six stages, each associated with a distinct area in the CNS. The first stage appears due to the lesions/dysfunction in the lower medulla oblongata. It includes subtle symptoms like unilateral resting tremors and changes in facial expression. The second stage ensues with damage to the raphe's lower nuclei, which manifests as motor symptoms affecting walking and posture. Stage 3 of the disease progresses to the substantia nigra, and patients begin to progress to motor symptoms such as difficulty balancing. The temporal mesocortex is affected in the fourth stage, followed by neocortical temporal fields. Many daily tasks are not possible, and even walking may need assistance. Finally, the cortex will be involved in stage 6, and patients are almost completely immobile and can have psychological manifestations such as hallucinations [2]. Although there are distinct stages of PD after diagnosis, initial symptoms of the disease (bradykinesia, resting tremor, and postural instability) are not present until approximately 70%-80% of dopaminergic neurons have been damaged [3]. Due to this fact, PD is considered a disease with a long latency period as the diagnosis is not likely to occur for years after the initial damage. It is, therefore, essential to diagnose PD as early as possible. The physical progression of the disease through the CNS is accompanied by a drastic worsening of symptoms and a decrease in treatment effectiveness [2].

Oxidative stress leading to dopaminergic neuron dysfunction in the substantia nigra has been considered the most plausible cause of PD [4,5]. Reactive oxygen species (ROS) can activate the caspase cascade in mitochondria, resulting in the cell's death [6]. Heavy metal poisoning, for example, often results in the accumulation of these toxins in the nigra material, resulting in reactive oxidative harm [2].

In addition, alpha-synuclein aggregation is a common finding in PD. These aggregations are harmful to dopaminergic neurons and may cause the formation of Lewy bodies (LB) and eventual necrosis [4]. The formation of LB can trigger a cascade of events. In a non-pathological state, LB aggregates are usually scavenged by a proteasome complex or lysosome. However, defects in these scavenging pathways are common in PD, which causes a further spread of aggregates [2]. LB are considered a defining pathological characteristic of PD and are also commonly found in dementia. It has been assumed that the initial alpha-synuclein travels through the vagus nerve, the major parasympathetic unit, from the enteric nervous system [2].

A cytochrome P450 2D6-deficient individual is nearly 2x more likely to develop PD in the presence of pesticides [2]. The normal function of this cytochrome is to metabolize pesticides, and the deficiency leads to the build-up of toxins. In addition, the presence of any ROS is likely to increase the risk of developing PD [2]. 

Antioxidants, natural sources, have recently gained popularity in combating the effects of ROS. The Zingiberaceae family contains the rhizome turmeric (*Curcuma longa*). For centuries, it has been used in India, China, and Southeast Asia for flavoring, food processing, coloring, and as traditional medicine [7]. Turmeric has long been used to treat rheumatism, eye infections, and liver problems [8]. Curcumin, turmeric's active ingredient, has antioxidant, anti-apoptotic, and anti-inflammatory properties that protect tissues from the harmful effects of ROS [9]. The phenol moiety, which donates a proton to ROS, is thought to be responsible for curcumin's antioxidant properties [8]. Curcumin also protects against A53T α-synuclein aggregation and monoamine oxidase B, becoming a compound of interest in treating neurodegenerative disorders such as PD [10,11]. Curcumin has been found to protect nigrostriatal dopaminergic neurons from damage in animal models. Curcumin had protective effects on alpha7-nicotinic acetylcholine receptors after administration of 6-hydroxydopamine (6-OHDA) in rats with a curcumin dose of 200 mg/kg [12]. Curcumin restored nigrostriatal dopamine neurons to 87.3% and 84.8% after low-dose 11-methyl-4-phenyl-1, 2, 3,6-tetrahydropyridine (MPTP) administration, compared to 49.1% in the MPTP group [3]. The use of tyrosine hydroxylase (TH) immunohistochemistry to determine dopamine denervation in coronal parts of the brain [12] further confirmed these findings [3].

The measurement of accurate biomarkers has become highly significant due to the long latent time between the onset of dopaminergic neuronal failure and PD symptom onset. Biomarkers to monitor the potential diagnosis of PD include neurochemical biomarkers and neuroimaging biomarkers. There are various risk factors associated with an increased likelihood of developing PD. A family genomic PD occurs earlier, but this accounts for only 10%-15% of all PD cases [2]. This indicates that a significant environmental factor plays a role in the pathology of PD. Any environmental factor that causes dopaminergic cell death may be considered a risk factor for developing PD. 

## Review

Prevalence of PD in different ethnic groups

PD is a global condition affecting people of all races and ethnicities. However, Wright et al. examined ethnic disparities and proposed that the prevalence of PD is higher in Caucasians than in African and Asian populations. As a result, there are known differences in PD incidence between Caucasians and Asians. Wright Willis et al. [13] found that Caucasian Americans had a higher incidence of PD than African Americans and Asians in a population-based study of Medicare recipients over 65 in the United States. In a study, Pringsheim et al. [14] observed a substantial difference in the prevalence of PD between Asia (646/100,000) and North America, Europe, and Australia (1601/100,000) in the population aged 70-79 years. According to these age-based studies, there is a variation in the prevalence of PD in different races at different ages.

Wright Willis et al. [13] support their claim with data from a population-based survey of over 65-year-olds in the United States conducted between 1995 and 2005, including over 450,000 PD cases per year. According to the findings, the prevalence of age-standardized PD (per 100,000) in white males was 2168.18 (95.64), 1036.41 (86.01) in blacks, and 1138.56 (46.47) in Asians. In a meta-analysis of the prevalence of PD by Pringsheim et al. [14], a significant difference in prevalence by geographical location and age (70-79 years of age) between 1985 and 2010 is noted. The results reported a prevalence of 1,601/100,000 in individuals from North America, Europe (including France, Italy, Spain, the Netherlands, and Germany), Australia, and South America (including Brazil, Uruguay, Argentina, and Bolivia), compared to a prevalence of 646/100,000 in individuals from Asia (including India, Taiwan, Hong Kong, Korea, China, Japan, Singapore, and Saudi Arabia) (P < 0), thus concluding that the prevalence of PD was much lower in Asia than in Europe, North America, and Australia. However, there is still a large variability in results in existing studies, so there is still much debate. This is due to other factors such as geographical location, cultural beliefs, and practices.

The data reported by Wright Willis et al. [13] and Pringsheim et al. [14] show that the highest prevalence of PD is in the white population, as with most existing studies. However, it is important to note [15] that other factors beyond ethnicity affect the prevalence of PD. They proposed that geographic area, rather than race, may be a more important determinant of PD prevalence. For example, the prevalence of PD in Black Africans in sub-Saharan Africa (40/100,000) is much lower than in people of African descent in the United States. In addition, the results of age-based studies may also be confused by cultural beliefs. For example, Dotchin and Walker [15] reported that many Chinese Americans viewed Parkinsonian symptoms as a consequence of aging, leading to delayed diagnosis. This could be a point of argument that PD prevalence is the same across ethnic groups. Nagashayana et al. [16] reported that the use of Ayurveda in Indian people impacts the presentation of PD symptoms and could potentially improve the outcome of the disease. Therefore, cultural practices also have a significant role in the prevalence of PD. In addition, Ben-Joseph et al. [17] noted that there is little public evidence of differences in the prevalence of PD in different ethnic groups that accommodate health inequalities, cultural practices, and geographical location. It is, therefore, imperative to note that while there is still evidence that PD claims are more prevalent in Caucasians than in the rest of the world, it is not yet sufficient in its bulk to make a firm conclusion. These differences among races should also alert healthcare providers when they are evaluating patients of different ethnicities as the appearance and presentation of disease may be variant. Providers must be cognizant of these variations to prevent missed diagnoses.

However, we cannot say that the difference is due exclusively to these two factors; we must also consider sociocultural differences. According to Dotchin and Walker [15], many Chinese Americans believe that Parkinsonian symptoms are a result of aging. This illustrates that different societies have different meanings of disease. As a result, there is a delay in diagnosis, and, as a result, the findings of age-based research are muddled. Furthermore, there are documented inequalities in access to advanced healthcare based on race and ethnicity [17]. As a result, the medical community needs to accept and investigate allopathic treatment practices as viable for treating conditions like PD. This is because they can have a higher uptake in some populations, reducing symptom incidence and disease progression. Nagashayana et al. [16], for example, found that the use of Ayurveda in Indians affects the presentation of PD symptoms and could potentially enhance the disease's outcome. This variation may be a result of the additional benefits of curcumin. 

Current allopathic treatments for PD**

Unfortunately, there is currently no curative treatment for PD. There are, however, a variety of ways to treat the symptoms and improve one's quality of life. Currently, both medications are designed to compensate for dopamine deficiency by either increasing dopamine levels, acting as dopamine agonists, or inhibiting dopamine metabolism. Common medicines include levodopa (L-dopa, L-3,4-dihydroxyphenylalanine), selegiline/rasagiline, entacapone/tolcapone, rapamycin, and adenosine A2A antagonists [2]. Surgery is a potential treatment, but it is used as a last resort when other methods are exhausted.

For this reason, it is only used in patients with highly advanced PD who are no longer able to manage their symptoms with drugs. Surgical intervention is a deep stimulation of the subthalamic nucleus of the brain [2]. Since advanced PD does not respond to levodopa, gene therapy for PD has been a developing area of research over the last decade. Target genes include aromatic amino acid decarboxylase (AADC) and glutamic acid decarboxylase (GAD) [2]. All of the traditional allopathic PD therapies have been designed to treat symptoms. Since they are less effective in treating advanced PD, we believe that a holistic approach could provide a better prognosis for these patients.

Levodopa

Tyrosine-based levodopa is a precursor to dopamine and is one of the most effective treatments for PD. Levodopa is converted to dopamine by the enzyme dopa decarboxylase.

However, this could be problematic because the enzyme could have decarboxylated orally administered levodopa before it reaches the CNS and would, therefore, not have been able to cross the blood-brain barrier. Carbidopa or benserazide is administered in conjunction with levodopa to ensure that it is not decarboxylated before the blood-brain barrier is crossed and the CNS is reached. Carbidopa and benserazide are classified as peripheral decarboxylation inhibitors. Carboxylated levodopa, combined with these inhibitors, can reach the CNS and decarboxylated to dopamine by serotonergic neurons [18,19]. 

Monoamine Oxidase (MAO) Inhibitors

MAO is the oxidative deamination and neurotransmitter degradation enzyme responsible for catecholamine families. Selegiline and rasagiline are included in this class. The dopamine metabolism can result in neuronal damage in dopaminergic neurons as a byproduct of oxidative deamination caused by the growth of ROS. However, those neurons are also protected against other ROS damage from dopamine metabolites by inhibiting the degradation of dopamine and not only by increasing dopamine function throughout the CNS [20].

Catechol o Methyltransferase (COMT) Inhibitors

COMT is a brain enzyme responsible for the inactivation of levodopa via methylation. Entacapone and tocapone inhibit COMT and thus prevent the inactivation of levodopa. These drugs may allow the levodopa dose to be effective for a more extended period of time [2,18].

Autophagy Upregulators

Part of the pathophysiology of PD is the accumulation of protein aggregates and LB. Autophagy refers to a cell's ability to destroy dysfunctional or pathogenic components. Rapamycin is a drug that can enhance the autophagy of neurons by inhibiting kinase mTOR (mammalian target of rapamycin). Therefore, the potential treatment of PD could be considered as reducing the accumulation of protein aggregates in the subthalamic nucleus [21].

Adenosine A2A

Adenosine A2A is a CNS receptor that antagonizes dopaminergic neurotransmission [22]. Adenosine A2A receptor antagonists such as caffeine have shown remarkable results in laboratory studies with transgenic mice and, more recently, in humans. Transgenic mice with mutant alpha-synuclein have been protected from PD if their adenosine A2A gene has also been removed [23]. Istradefylline has shown tremendous promise in reducing "OFF" time in PD patients. "OFF" time is considered to be the period during which PD patients return their motor symptoms and dyskinesia. Generally, "OFF" time increases the longer the patient has PD, more specifically, the longer the patient has been treated with levodopa [22,24]. Therefore, the combination of istradefylline and levodopa therapy is likely to reduce "OFF" time in advanced PD patients effectively. 

Deep Brain Stimulation (DBS)

DBS is considered only when PD symptoms are extremely advanced and can no longer be controlled adequately with oral medication. Generally, DBS targets the subthalamic nucleus through an electrical stimulator using radiologically guided intracranial electrodes [25]. The diseased neuronal pathways would be either excited or inhibited by this electrical excitement. The release of dopamine could be activated through this process [25]. However, the risk of post-DBS infection and waiting time for treatment are high for PD surgery [2].

Gene Therapy

Patients with PD have shown a decrease in AADC, leading to less conversion of levodopa (L-DOPA) to dopamine. Because of this, the upregulation of this gene combined with sufficient levodopa intake would be beneficial for PD symptoms [2]. GABA is a neurotransmitter inhibitor. GAD helps GABA-ergic neurons produce more GABA. The lack of dopamine in PD triggers a chain of events that result in unnecessary muscle contractions and motor symptoms. These symptoms could be reduced by the upregulation of GAD and the subsequent increase in the inhibitory GABA neurotransmitter [26]. 

An alternative approach: curcumin and its neuroprotective effects**

Curcumin's Mode of Action

Curcumin's protective properties start with its ability to cross the blood-brain barrier due to its lipophilic nature [27]. Curcumin has various protective properties in the brain, including protection against toxic metals and ROS. Toxic metal ions can interfere improperly with tissues in the brain, causing neurological damage. Curcumin, as a flavonoid, has antioxidant properties that are potentially stronger than typical antioxidants such as vitamins C and E [3]. The brain is more susceptible to oxidative damage than other body tissues because it absorbs a higher percentage of oxygen (around 20%) than other tissues. With too much oxygen, the formation of ROS such as peroxide accumulates over time, resulting in lower mitochondrial density, lower overall ATP output, and a decreased ability to sustain intracellular ion concentrations, ultimately leading to neuron death. Curcumin's ability to donate an H ion from the beta-diketone moiety is thought to be responsible for its anti-ROS properties [28]. Curcumin protects mitochondria and neurons from the damaging effects of ROS by donating an H ion. The development of LB is related to the onset of PD. Alpha-synuclein oligomers clump together to form LB. Curcumin has been shown to prevent alpha-synuclein oligomer aggregation [28]. 

Protecting Effects of Curcumin in Animal Atudies

In one study, intrastriatal 6-OHDA injections were administered to rats to induce parkinsonism. One group received 200 mg/kg of curcumin over four weeks, but not the other. A reverse response to cognitive impairment was used to determine. Average control groups over the 30-minute test averaged 8.9 ± 5 turns. The rats treated with 6-OHDA had, on average, 257.8 ± 23.4, which was considerably superior to control. There has been a significant reduction in turns with just 126.9 ± 23.8 turns in the group administered with 6-OHDA and curcumin during the 30-minute test. Following the experiment, TH antibodies stained the brains of the test animals. The staining density was used to determine the amount of fibers that produced dopamine left after each treatment.

In contrast to the control group, the curcumin rat kept 32.46% ± 4.2% of its fibers (98.29% ± 5.9%). In the group with 6-OHDA without curcumin, the control was only 7.14% ± 3.2% [29]. In another animal study, MPTP administration of parkinsonism was applied to rats. MPTP was given to the first group only, MPTP + 1 mg/kg of curcumin to the second group, and MPTP + 2 mg/kg of curcumin to the third group. All test groups were assessed the total movement distance in 10 minutes. The group treated with MPTP alone had a 32.0% decrease in movement over control. The MPTP + 1 mg/kg curcumin-treated trial group only saw a 59.4% increase, with MPTP + 2 mg/kg curcumin movement increasing by 136% over the control group. The experiment involved taking brain sections and the analysis of TH antibody expression. The group without curcumin but administered MPTP experienced an increase to 42.9% from the control of TH expression. The MPTP + 1 mg/kg group of curcumin has increased to 60.3%, and the MPTP + 2 mg/kg of curcumin has increased to 74.8% compared to the control group. The dose-dependent response of curcumin has become clear in this study [30].

Limitations of the study

The differences in the prevalence of PD among different ethnicities are reported in many studies. Researchers have found that genetic factors, geographical location, and cultural practices all play a significant role in the presentation, diagnosis, and management of this complex disease. Though we speculate curcumin consumption as a significant determinant of the observed differences in the prevalence of PD, future studies directly comparing the dosage with the prevalence of PD would provide unequivocal evidence for the protective role of curcumin in PD. Current allopathic treatments are discussed in Table 1.

**Table 1:** Current Allopathic Treatments for Parkinson's Disease. MAO: monoamine oxidase; COMT:  Catechol o methyltransferase; RBC: red blood cells; WBC: white blood cells; A2A: adenosine A2A antagonists; AADC: aromatic amino acid decarboxylase; L-DOPA: levodopa.

Allopathic Treatment	Mechanism of Action	Adverse Effects
Levodopa	Dopamine precursor (given with carbidopa to decrease peripheral metabolism)	Hallucinations, anxiety, depression, cardiac arrhythmias
MAO inhibitors	Inhibition of dopamine metabolism and deamination	Serotonin syndrome and hypertensive crisis when used with serotonergic drugs
COMT inhibitors	Inhibition of dopamine inactivation and methylation	Tolcapone can lead to hepatic necrosis
Autophagy upregulators	Enhance neuronal ability to degrade dysfunctional proteins	Pancytopenia (decrease in RBC, WBC, and platelet counts)
Adenosine A2A	Blockade of dopaminergic inhibition	Hallucinations, muscle spasms, insomnia, nausea, vomiting
Deep brain stimulation	Electrical stimulation of the subthalamic nucleus	Seizures, infection at site of entry, stroke, headache
Gene therapy	Upregulation of AADC, leading to increased conversion of L-DOPA to dopamine	Immune reactions

## Conclusions

This review examined the use of curcumin, the active ingredient in turmeric, to improve PD therapy and outcomes. Diet and lifestyle play a role in PD pathogenesis, but their role is unclear. Current therapy strategies include levodopa combined with MAO inhibitors, COMT inhibitors, A2A antagonists, and autophagy upregulators. In circumstances where drugs fail to work, surgery is used. The fact that PD is less common in South Asian nations like India supports the idea that lifestyle and nutrition choices may help delay onset or development. Turmeric's active element is curcumin, which is consumed in up to 200 mg daily doses in Indian cuisine. Curcumin's powerful antioxidant capabilities, including chelation of harmful metals and lowering ROS, may help reduce inflammation and apoptosis. Because neurons are terminal tissues, reducing ROS may improve their longevity and the efficacy of pharmaceutical therapies. This paper first summarizes existing PD therapies. Next, we examine curcumin's antioxidative capabilities and its chemistry. Finally, we examined research that shows curcumin protects dopaminergic neurons in PD animal models. These findings imply that curcumin could be used in conjunction with standard PD medication to improve treatment and results. Future research on curcumin in PD treatment will help uncover its potential benefits in reducing disease progression and promoting recovery.
